# Multi-atlas segmentation of subcortical brain structures via the AutoSeg software pipeline

**DOI:** 10.3389/fninf.2014.00007

**Published:** 2014-02-06

**Authors:** Jiahui Wang, Clement Vachet, Ashley Rumple, Sylvain Gouttard, Clémentine Ouziel, Emilie Perrot, Guangwei Du, Xuemei Huang, Guido Gerig, Martin Styner

**Affiliations:** ^1^Department of Psychiatry, University of North CarolinaChapel Hill, NC, USA; ^2^Scientific Computing and Imaging Institute, University of UtahSalt Lake City, UT, USA; ^3^BioclinicaLyon, France; ^4^Department of Neurology, Neurosurgery and Radiology, Pennsylvania State University Milton Hershey Medical CenterHershey, PA, USA; ^5^Department of Computer Science, University of North CarolinaChapel Hill, NC, USA

**Keywords:** segmentation, registration, MRI, atlas, brain, Insight Toolkit

## Abstract

Automated segmenting and labeling of individual brain anatomical regions, in MRI are challenging, due to the issue of individual structural variability. Although atlas-based segmentation has shown its potential for both tissue and structure segmentation, due to the inherent natural variability as well as disease-related changes in MR appearance, a single atlas image is often inappropriate to represent the full population of datasets processed in a given neuroimaging study. As an alternative for the case of single atlas segmentation, the use of multiple atlases alongside label fusion techniques has been introduced using a set of individual “atlases” that encompasses the expected variability in the studied population. In our study, we proposed a multi-atlas segmentation scheme with a novel graph-based atlas selection technique. We first paired and co-registered all atlases and the subject MR scans. A directed graph with edge weights based on intensity and shape similarity between all MR scans is then computed. The set of neighboring templates is selected via clustering of the graph. Finally, weighted majority voting is employed to create the final segmentation over the selected atlases. This multi-atlas segmentation scheme is used to extend a single-atlas-based segmentation toolkit entitled AutoSeg, which is an open-source, extensible C++ based software pipeline employing BatchMake for its pipeline scripting, developed at the Neuro Image Research and Analysis Laboratories of the University of North Carolina at Chapel Hill. AutoSeg performs N4 intensity inhomogeneity correction, rigid registration to a common template space, automated brain tissue classification based skull-stripping, and the multi-atlas segmentation. The multi-atlas-based AutoSeg has been evaluated on subcortical structure segmentation with a testing dataset of 20 adult brain MRI scans and 15 atlas MRI scans. The AutoSeg achieved mean Dice coefficients of 81.73% for the subcortical structures.

## Introduction

Accurate segmentation of brain structures from magnetic resonance imaging (MRI) (Khan et al., 2011), functional MRI (fMRI) (Maldjian et al., 2003) and positron emission tomography (PET) (Tohka et al., 2007) is essential for quantitative studies of the brain, such as disease progression and aging. In general, manual brain anatomical labeling (identification of anatomical brain structures and assignment of a unique label to each structure) is considered the most accurate means of giving the most accurate results closest to the true segmentation of brain structures. However, as the size and availability of large MRI databases increase, manual segmentation of brain structures is not realistic means of segmenting the brain because of the significant time-cost of human raters and un-predictable intra- and inter-rater variability. Therefore, automated segmentation methods are highly desirable when the size of MRI databases is considerably large (e.g., >50 cases). However, automated anatomical brain region segmentation (labeling) of subcortical regions in MRI data is challenging, since the contrast between tissues is often low for a variety of brain structures (“Subcortical regions” is included since folding/shape variation may be a bigger determining factor than contrast for labeling cortical structures.). The commonly present shape and intensity variations in a number of diseases further complicate robust brain segmentation.

Atlas-based segmentation is a simple method for automated segmentation as it is a compromise between manually driven and fully automated segmentation approaches (Bajcsy et al., 1983; Gee et al., 1993; Collins et al., 1995; van Leemput et al., 1999b). In atlas-based segmentation methods, image information (intensity and spatial) is transferred from the labeled atlas to subjects through non-rigid image registration. Thus, the performance of the registration algorithm would have a big impact on the accuracy of the final segmentation. Because the image registration algorithms are inherently related to the anatomical similarity between atlas and subject, an atlas that is anatomically similar to a subject would result in better performance for the segmentation. Therefore, a good choice of atlas can help in accurately segmenting the majority of images. However, as inherent anatomical variability may present in most brain MRI data sets, the choice of atlas for improving the segmentation performance will likely under-perform on outlying images presenting abnormal pathologies.

Multi-atlas-based segmentation has shown the potential to resolve this issue by using a number of atlases with differences in anatomy as the atlas population, performing multiple non-rigid registrations from all the labeled atlases to the subject and fusing the propagated labels to generate the final segmentation (Rohlfing et al., 2004; Warfield et al., 2004; Heckemann et al., 2006; Wang et al., 2012; Asman and Landman, 2013). By use of multiple atlases in this way, better segmentation results can be expected, because the anatomical variability is represented more accurately than in a single atlas. Furthermore, the errors due to inaccurate labeling or registration can also be averaged out, when the individual propagated labels are fused together (Asman and Landman, 2011). Label fusion generally plays an important role in the multi-atlas-based segmentation approaches. It is achieved using a majority voting in the simplest case and much of the current research is focused on improving the label fusion step (Warfield et al., 2004; Isgum et al., 2009; van Rikxoort et al., 2010; Wang et al., 2012; Asman and Landman, 2013). A limitation of the multi-atlas-based segmentation methods is that the individual differences that occur in only a minority of the atlases could be averaged out. Thus, the segmentation results would be biased, particularly for the abnormal MRI scans with pathologies. In order to address this issue, appropriate atlas selection is needed.

One example of atlas selection is the use of atlas-subject registration accuracy estimators to weight the influence of a given atlas (Wu et al., 2007; Artaechevarria et al., 2009; Isgum et al., 2009; van Rikxoort et al., 2010). Similarly, methods that employ image similarity metrics, such as mutual information, to select atlases (Aljabar et al., 2009) are also examples of atlas selection, which presume that choosing those atlases whose registered images are similar to the subject will result in more accurate segmentations. However, these approaches could not handle the registration with large initial dissimilarity in shape between the atlases and the target. This can lead to inappropriately high weights in cases of initially large shape differences resulting in incorrect image correspondences established by the atlas registration.

Recently, several segmentation methods using graph-based (Hamm et al., 2010; Jia et al., 2012a) or tree-based (Jia et al., 2012b) intermediate templates guided registration methods have been demonstrated to be effective in the segmentation of brain MR images. The key concept of these methods is to decompose a large deformation into several small deformations that can be estimated with higher reliability. Each atlas is warped through the intermediate templates one by one on the path toward the subject. However, a major problem of the above strategy is that the quality of the warped atlas will be affected by accumulated registration errors. Similar to these approaches Langerak and Berendsen (2013) proposed a multi-atlas segmentation method with pre-registration atlas selection. The atlas set was clustered (Frey and Dueck, 2007) and exemplars for each cluster were selected to generate a preliminary segmentation of the subject using a majority voting label fusion. The cluster with the highest similarity to the preliminary segmentation was selected to create the final segmentation of subject. While this method is somewhat close to the proposed method here, it assumes that the difference between preliminary segmentation and true segmentation is minor, which is not necessarily guaranteed. Furthermore, this method ignores the intensity information in the target image and the atlas images. Finally, any sample bias in the multi-atlas population that could bias a subsequent segmentation is further aggregated by employing only the closest or best cluster. In contrast, we proposed a novel atlas selection method that makes use of all clusters with each one only contributing a single exemplar atlas, the one closest to the subject image.

In this study, we proposed a multi-atlas-based segmentation scheme with a novel graph-based atlas selection technique. We first register all atlases to the subject MR scan. The atlases are also paired and co-registered with each other. A directed graph with edge weights based on intensity and shape similarity between all MRI scans is then computed. In contrast to the atlas selection strategies discussed above, we proposed a novel atlas selection method that separates the graph into several clusters and makes use of all clusters with each one only contributing a single exemplar atlas (neighboring template), the one closest to the subject image. Finally, weighted majority voting is employed to create the final segmentation over the selected neighboring templates. We use this multi-atlas-based segmentation scheme to extend a single-atlas-based segmentation toolkit entitled AutoSeg, which is an open-source, extensible C++ based software pipeline developed at the Neuro Image Research and Analysis Laboratories (NIRAL) of the University of North Carolina at Chapel Hill. This software pipeline employs BatchMake pipeline scripts that call tools within the AutoSeg toolset based on the Insight Tool Kit (ITK). AutoSeg has been used and is in use in a number of studies, including Parkinson's disease (Lewis et al., 2009; Du et al., 2011, 2012; Sterling et al., 2013), autism (Hazlett et al., 2009, 2011, 2012), schizophrenia (McClure et al., 2013), craniosynostosis (Paniagua et al., 2013), and drug abuse (Gerig et al., 2011). AutoSeg entails intensity inhomogeneity correction, brain tissue classification based skull-stripping, rigid and non-rigid image registration, and multi-atlas-based segmentation with atlas selection. For the multi-atlas-based segmentation step, an atlas population consisting of multiple brain MRI scans and corresponding structural region of interest (ROI) (label files) and/or lobar subdivision definitions (parcellation files) is needed as input. Through its transparency in atlas definitions, parameter definitions, and enabling/disabling of individual processing steps, all saved within designated preference files, AutoSeg is highly adaptive and thus designed for use in all ages including young neonates, adolescent, adult, and even elderly populations. Please note that while AutoSeg enables the use of multiple (two) modalities, it is still a mono-modal scheme, as it aims at co-registering images only of the same modality, such as a joint weighted T_1_ to T_1_ weighted and T_2_ to T_2_ weighted registration.

## Materials and methods

### Materials

We evaluated the proposed multi-atlas segmentation within AutoSeg with a dataset of 35 defaced T_1_-weighted structural MRI scans. Fifteen scans (5 males and 10 females with an age range of 19–34) were used as the multi-atlas population and the remaining 20 scans (8 males and 12 females with an age range of 18–90) were used for testing. Thus, the 20 testing MRI scans were segmented one-by-one using the 15 atlases. These MRI scans were selected from the Open Access Series of Imaging Studies (OASIS) database (http://www.oasis-brains.org) (Asman and Landman, 2013). This dataset has been used in the MICCAI 2012 Multi-Atlas Labeling challenge, URL: https://masi.vuse.vanderbilt.edu/workshop2012/. This dataset was expertly labeled courtesy of Neuromorphometrics, Inc. (Somerville, MA) and provided under a non-disclosure agreement of the Creative Commons Attribution-NonCommercial (CC BY-NC). For each atlas, a collection of 28 labels of subcortical structures were used (Asman and Landman, 2013): 3rd ventricle, 4th ventricle, brain stem, left/right hemispheric accumbens, cerebral White Matter (WM), cerebellar WM, caudate, amygdala, hippocampus, lateral ventricle, pallidum, putamen, thalamus, and ventral diencephalon (DC), as well as cerebellar vermal lobules I-V, VI-VII, and VIII-X. All images are 1 mm isotropic resolution.

### Method summary

**Figure 1:**
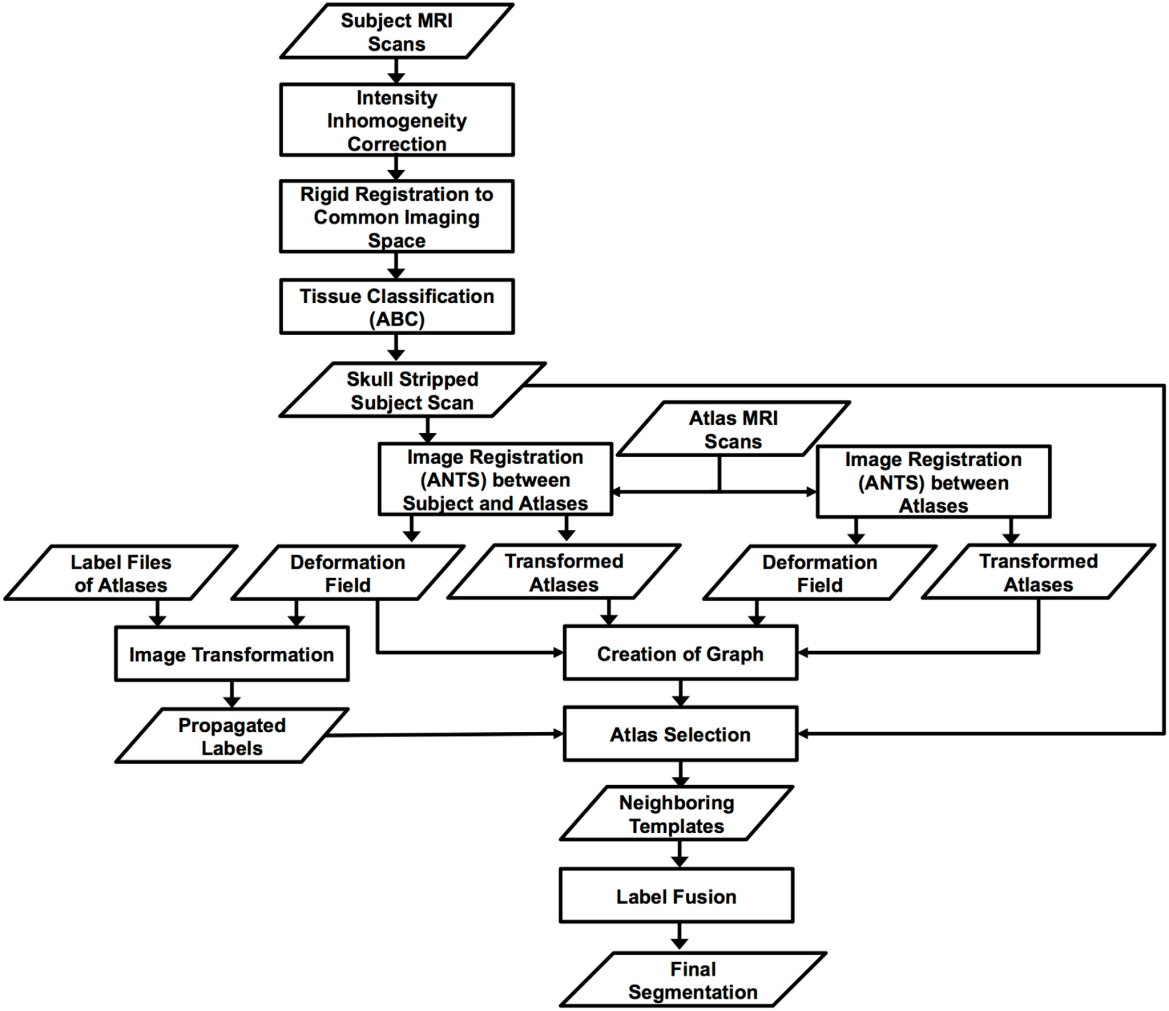
**Overall computational scheme of AutoSeg with multi-atlas segmentation**.

In summary, we extended a single-atlas-based segmentation toolkit entitled AutoSeg, with an additional multi-atlas-based segmentation tool. The processing pipeline of the proposed method is shown in Figure 1. The AutoSeg software pipeline is publicly available under a BSD license on the NITRC website, at http://www.nitrc.org/projects/autoseg. AutoSeg starts with intensity inhomogeneity correction, followed by registration into a common MRI template space (such as standard MNI space). Next, the Atlas Based Classification (ABC) tool is applied to perform atlas moderated, Expectation Maximization based tissue classification (van Leemput et al., 1999b; Prastawa et al., 2003) for skull-stripping. AutoSeg then employs the symmetric diffeomorphic registration via the ANTS (Advanced Normalization ToolS) registration tool (Avants et al., 2008) to align all atlases and subject MRIs with a non-rigid diffeomorphic image registration scheme. The label files for each atlas are warped with the computed deformation field from the atlases to the subject data. A fully connected graph is then constructed, including all the atlases and the subject image. Every edge between two vertices of the graph is assigned a cost, which is defined by a weighted sum of an intensity similarity term and a shape similarity term. We cluster the atlas population into groups by searching the shortest path from each atlas to the subject. Atlases on the same shortest paths are combined into the same cluster. We then select the atlas that is closest to the subject for each cluster as the neighboring template. Finally, the propagated label files of the neighboring templates are fused to create the final segmentation via a weighted majority voting label fusion. In summary, the major results of AutoSeg include (a) the bias-corrected, atlas co-registered, skull-stripped MR images, (b) a tissue classification with optional parcellation, and (c) the multi-atlas-based regional segmentations. In the following sections, we discuss each step of AutoSeg's segmentation framework in more detail.

### Intensity inhomogeneity correction

Intensity inhomogeneity in MRI is typically caused by the imperfections of the image acquisition process, such as B1 inhomogeneity, receive coil non-uniformity or static field inhomogeneity (Hou et al., 2006; Vovk et al., 2007). We employ the N4 algorithm (Tustison et al., 2010) to correct intensity inhomogeneity in AutoSeg. N4 is an extension of the well-known N3 algorithm (Sled et al., 1998) that has been routinely used in many MRI-based studies and applications. This iterative method determines a multiplicative smooth field that maximizes the high frequency content of the tissue intensity distribution.

### Registration into common template space

After intensity inhomogeneity correction, each subject's MRI scan (commonly a T_1_ weighted and optionally a T_2_ weighted scan per subject) is rigidly aligned to a common space of an existing brain atlas, usually a template image in the ICBM atlas space (Mazziotta et al., 2001). We use rigid registration to align the subject MRI scans to a common space, because it has the advantage that the input images are not distorted and thus measurements made in that space do not need to be adjusted. The registration is done using a standard rigid transformation with a normalized mutual information based metric. This is achieved via the 3D BRAINSFit (Johnson et al., 2007) tool within 3D Slicer [called “General Registration (BRAINS)” in Slicer's User Interface].

### Atlas based classification (ABC) and skull-stripping

Skull-stripping or whole brain segmentation refers to the processing of separating the brain tissues [gray matter (GM), white matter (WM) and cerebrospinal fluid (CSF)] from non-brain image parts such as, sclera, orbital fat, skin, etc. It is an important step of many neuroimaging applications, such as surgical planning, cortical surface reconstruction and brain morphometry, which depend on the ability to accurately segment brain from non-brain tissue. In this study we employ the Atlas Based Classification (ABC) tool (van Leemput et al., 1999a,b; Prastawa et al., 2003) to perform tissue segmentation as well as skull-stripping integrated into a single method.

**Figure 2:**
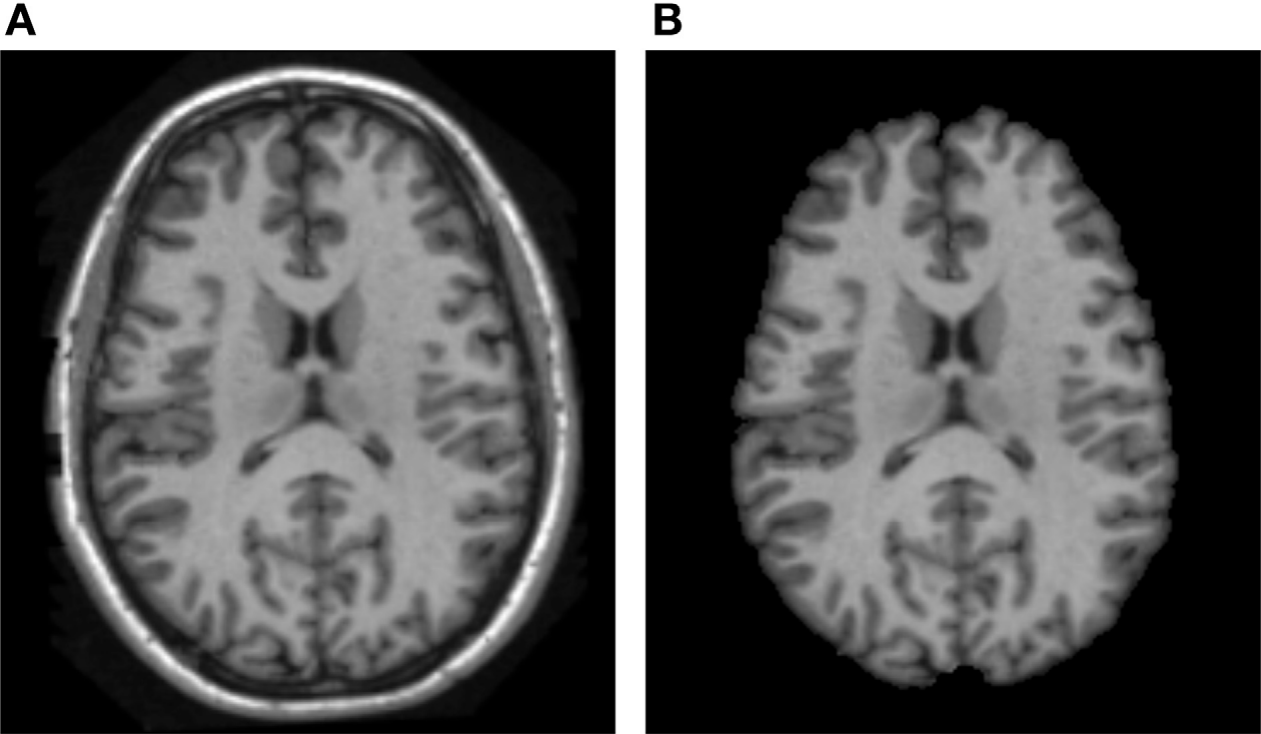
**ABC based brain skull-stripping result. (A)** the brain tissue in original MRI scan, **(B)** the skull-stripped brain.

ABC is ITK-based and can be run within 3D Slicer or as a stand-alone tool. ABC classifies brain MRI voxels into GM, WM, and CSF via standard atlas moderated Expectation-Maximization (EM) optimization (van Leemput et al., 1999b) and an atlas template mapping using fluid image registration (Christensen et al., 1996). The hard tissue segmentations are then combined, smoothed via mathematical morphology operations and level-set based smoothing (Styner et al., 2006), and hole filled to create a brain tissue mask. Figure 2 show an example of skull-stripped image of the scan using ABC.

### Multi-atlas-based segmentation with atlas selection

#### Image registration

As mentioned before, non-rigid registration plays an indispensable role in the atlas based, particularly multi-atlas-based segmentation approaches. In AutoSeg we employ the ANTS registration tool (as part of the ANTS registration package) (Avants et al., 2008) to register each skull-stripped atlas image to the skull-stripped subject image using a cross-correlation similarity metric and a symmetric diffeomorphic deformation model that preserves anatomical topology even with large deformation. We use cross-correlation as the image registration metric within ANTS, due to its enhanced reliability and accuracy over mean square error in our experiments. Furthermore, in Klein et al. (2009) mean squared error based registration algorithms performed significantly worse than cross-correlation based ANTS, though cross-correlation was not compared directly with mean squared error in the same registration algorithm. One additional advantage is that it does not require intensity calibration between the target and the source images. The transformation is differentiable and guaranteed to be smooth and one-to-one, i.e., for every element in the moving image, there is a single corresponding element in the fixed image. The transformation field obtained from the registration is then employed to propagate the brain labels of each atlas. Prior to the atlas-to-subject image registrations, all atlases are like-wise co-registered with each other, i.e., each atlas is pairwise registered to all the other atlases.

#### Construction of graph

**Figure 3:**
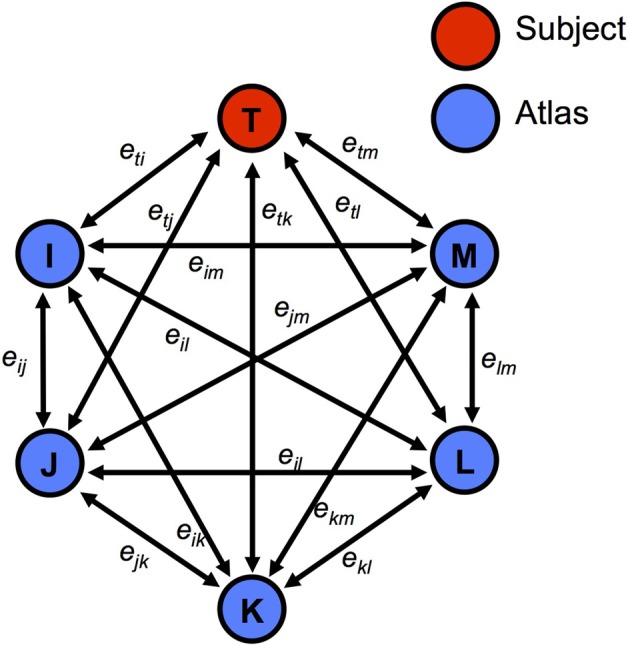
**Example of a graph with the subject T and atlases I, J, K, L and M**. The graph is constructed based on the similarity measurements between image pairs.

We represent the registered dataset as a graph (Figure 3) whose vertices correspond to the atlases and target. Every edge between two vertices is assigned a cost (*e*_*ij*_), which is defined by a weighted sum of an intensity similarity term M_*ij*_ (mean squared voxel-wise intensity difference) and a shape similarity term H_*ij*_ (harmonic energy) [Equation (1)].

(1) $${\text{e}}_{ij}=w_{1}{\text{M}}_{ij}+w_{2}{\text{H}}_{ij}$$

where *w*_1_ and *w*_2_ represent the weighting factors for the intensity similarity term and shape similarity term, respectively. We empirically determined a combination of *w*_1_ = 0.2 and *w*_2_ = 0.8 to be the weighting factors. The weighting factors were determined by applying the AutoSeg to a training dataset, i.e., a small group of MRI scans, and then selecting the combination of parameters that produced the best segmentation of the training dataset. The *M* intensity difference is defined by

(2) $${\text{M}}_{ij}=\frac{1}{N}\sum_{m=1}^{N}{\left(i_{m}-j_{m}\right)}^{2}$$

where *i*_*m*_ is the intensity of *m*-th voxel of a MRI scan *I*; *j*_*m*_ is the intensity of *m*-th voxel of another MRI scan *J*, where *J* is registered to *I*. *N* is the number of voxels in a MRI scan.

The shape similarity term *H* is defined as the harmonic energy, which is the mean Frobenius norm of the Jacobian of the deformation field from ANTS registration (Hamm et al., 2010).

#### Clustering-based atlas selection

**Figure 4:**
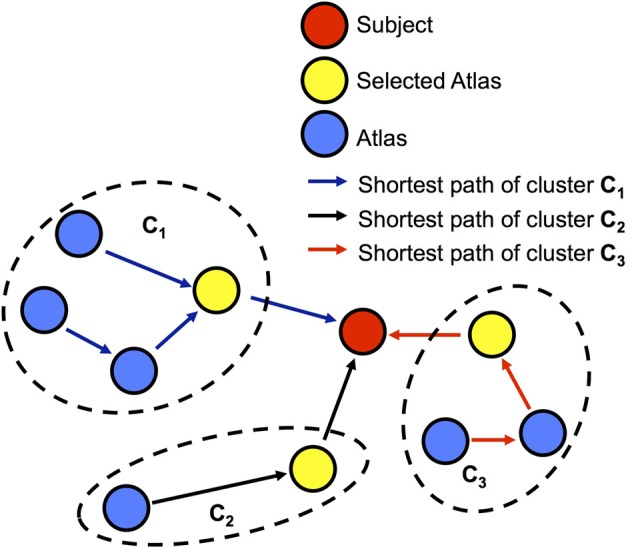
**Clustering-based atlas selection framework**.

From the graph constructed in the previous section, we can choose atlases that are close to the subject via an atlas clustering. On this graph, we cluster the atlas population into groups by searching the shortest path from each atlas to the subject using the Floyd-Warshall algorithm (Floyd, 1962). We assume that the atlases on the same shortest path belong to the same cluster. We then select the atlas that is closest to the subject in each cluster as the neighboring template for the final segmentation. An example of the clustering from a graph is illustrated in Figure 4 to demonstrate the framework of the atlas selection. In this example, the atlases are partitioned into three clusters. Three neighboring templates are selected for creating the final segmentation of the subject. It is noteworthy that the clustering result changes for every subject image, i.e., the atlases cannot be pre-clustered in this approach.

#### Weighted majority voting label fusion

Majority voting is the most widely used label fusion algorithm for multi-atlas-based segmentation approaches. This algorithm weights each candidate segmentation equally and assigns to each voxel the label on which most segmentations agree (Heckemann et al., 2006). However, the assigned label by this simple majority rule does not necessarily imply a correct segmentation in applications with large variation in size, shape, and appearance. This issue can be mitigated via a weighted majority voting approach, i.e., assigning larger weights to the atlases that show higher similarity to the subject image. For each selected neighboring template, we use one minus the edge cost between that template and the subject on the graph as its voting weight. The final segmentation is determined by collecting weighted votes from all the segmentations over the selected neighboring templates and assigning the label with the highest vote to each voxel.

### Segmentation performance assessment

We assess the performance of our proposed segmentation method by evaluating how close the resulting segmentation is to the corresponding reference segmentation. The most commonly used metric is the Dice similarity coefficient (DSC), also referred to as the mean overlap or the similarity index, which is computed between two segmentations as:

(3) $$DSC=2\times \frac{V_{\text{auto}}\cap V_{\text{ref}}}{V_{\text{auto}}+V_{\text{ref}}}\times 100\%$$

where *V*_auto_ and *V*_ref_ are the volume of the automated segmentation result and the volume of the reference segmentation, respectively. A DSC of 1 indicates complete volumetric overlap, and 0 indicates no overlap at all. We also employ the symmetric mean absolute distance (MAD) and Hausdorff distance (Wang et al., 2009) between the surfaces of the resulting segmentation and the corresponding reference segmentation as additional metrics to evaluate the segmentation results. MAD is calculated by measuring the average distance from all points on the surface of the automatically segmented brain tissue to the surface of the reference segmentation. On the other hand, to assess the maximal local discrepancy between an automatic segmentation and reference segmentation, the symmetric Hausdorff distance between the surface of the automatically segmented brain tissue and that of the reference segmentation is calculated. The smaller the MAD or Hausdorff distance, the better aligned the points on the two surfaces and thus the better the agreement with the reference segmentation.

## Experimental results

**Figure 5:**
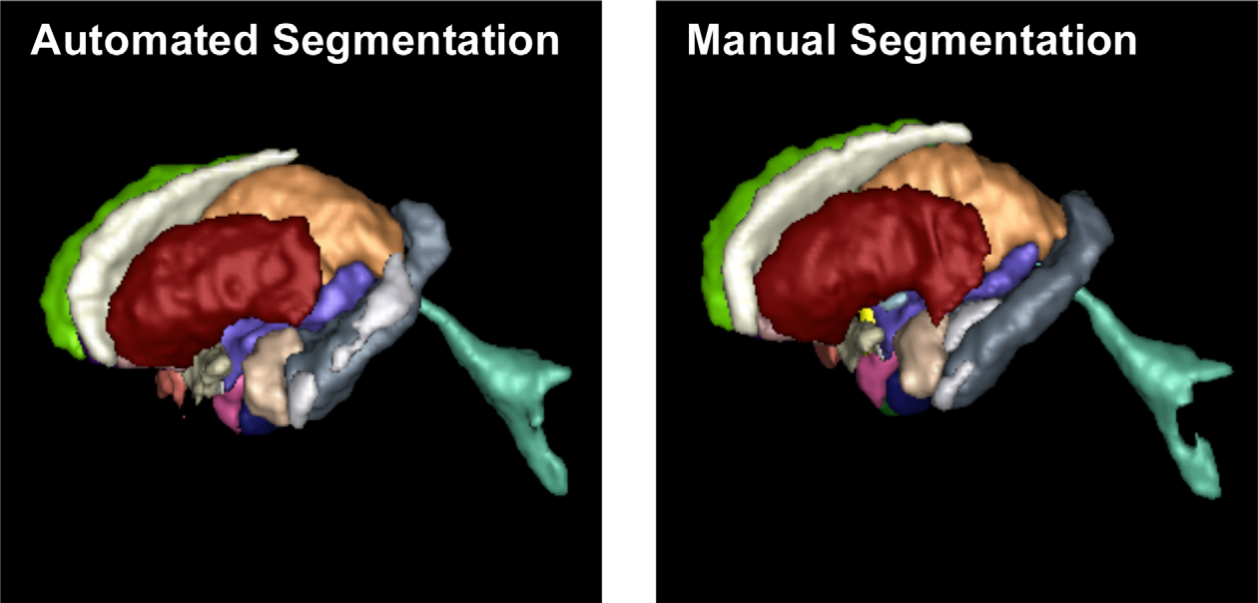
**Visual comparison between structures segmented by AutoSeg (left) and the corresponding manually segmented structures (right) via 3D rendering**.

We have applied the AutoSeg segmentation software pipeline to the brain MRI data set with 20 testing scans and 15 atlases. The parameter settings of this experiment are described in the Appendix. Table 1 summarizes the mean values of the DSC, MAD, and Hausdorff distance of the 28 subcortical structures for the 20 testing MRI scans in our database. The mean DSC, mean MAD and the mean Hausdorff distance for subcortical regions were 81.73%, 0.57 and 5.68 mm, respectively. All structures showed a MAD below 1 mm, which indicates sub-millimeter accuracy on average (at a 1 mm isotropic image resolution). Smaller, skinnier structures showed DSC above 70% and larger structures were all above 80% DSC. Figure 5 shows the 3D segmentation results of subcortical structures on a selected example. The segmentation results and the parameter settings of the testing data set used for our experiment are available at the NITRC web page of AutoSeg: http://www.nitrc.org/docman/view.php/421/1312/MICCAI_2012_Challenge_Data_Seg.zip.

**Table 1:** **Mean Dice Similarity Coefficient (DSC), symmetric Mean Absolute Distance (MAD), and symmetric Hausdorff distance for subcortical structures**.

	**DSC (%)**	**MAD (mm)**	**Hausdorff distance (mm)**
3rd Vent	73.34±5.56	0.53±0.18	5.1±2.3
4th Vent	79.37±3.64	0.52±0.23	7.87±3.96
Right accumbens	70.32±8.34	0.55±0.22	4.18±2.02
Left accumbens	70.81±7.83	0.54±0.2	3.81±2
Right cerebral WM	87.75±2.05	0.49±0.09	7.71±2.16
Left cerebral WM	87.31±1.82	0.5±0.08	9.42±4.33
Right cerebellum WM	86.02±3.47	0.57±0.25	8.25±2.26
Left cerebellum WM	86.13±3.89	0.57±0.29	8.81±2.61
Brain stem	90.46±1.65	0.55±0.15	6.85±3.53
Right caudate	75.2±13.98	0.76±0.51	5.37±2.75
Left caudate	74.68±16.87	0.82±0.69	5.35±3.4
Right amygdala	75.92±2.99	0.56±0.08	3.96±0.93
Left amygdala	76.93±2.93	0.55±0.09	3.33±1.09
Right hippocampus	79.03±3.64	0.59±0.16	5.39±1.63
Left hippocampus	80.64±2.55	0.56±0.14	6.51±2.12
Right lateral ventricle	83.46±4.79	0.61±0.24	9.65±5.39
Left lateral ventricle:	84.01±3.91	0.6±0.29	7.86±3.03
Right pallidum:	83.8±4.5	0.42±0.07	2.71±0.63
Left pallidum:	84.3±2.06	0.41±0.05	2.76±0.56
Right putmen:	88.02±3.08	0.38±0.08	3±0.89
Left putmen:	88.11±3.58	0.38±0.1	3.23±1.21
Right thalamus	89.5±2.06	0.51±0.1	4.01±1.71
Left thalamus	89.32±2.05	0.53±0.1	4.19±1.71
Right ventral DC	85.02±1.99	0.53±0.1	5.53±2.97
Left ventral DC	84.84±1.97	0.54±0.11	5.25±3.03
Cerebellar vermal lobules I-V	78.32±3.76	0.84±0.2	7.27±7.58
Cerebellar vermal lobules VI-VII	72±4.95	0.89±0.3	6.88±2.9
Cerebellar vermal lobules VIII-X	83.7±4.39	0.58±0.32	4.89±3.2

The most time consuming steps of our segmentation method are the ANTS based image registration step and the ABC based brain tissue classification. The average computational time of the ANTS registration of one pair of images on a standard workstation with 2.6GHz CPU (running on as a single core/thread) and 8GB RAM was 185 min; the average computational time of the ABC tissue classification including deformable, fluid image registration was 48 min. The remaining steps were performed with an average computational time less than 5 min.

## Discussion

In this paper we present both a novel label fusion algorithm for multi-atlas-based brain segmentation, as well as a comprehensive, extendable brain segmentation software pipeline called AutoSeg. For the label fusion, we employ an approach that incorporates shape and intensity information of subject and atlases for both atlas selection and fusion weighting.

The multi-atlas-based AutoSeg segmentation software pipeline was tested on a dataset with 15 atlases and 20 testing MRI scans for the segmentation of subcortical structures. As volumetric analysis of subcortical structures is a major aim in various neuroimaging studies, many automated segmentation methods, particularly atlas-based methods, have been developed as mentioned in the introduction section. Liu et al. (2007) and Gouttard et al. (2007)'s single atlas-based methods achieved mean DSC of 74.66 and 79%, respectively, over the major subcortical structures (caudate and putamen for Liu's study and amygdala, caudate, hippocampus, lateral ventricle, pallidus, and putamen for Gouttard's study) in different settings. In our multi-atlas-based method, we achieved a mean DSC of 81.50% for caudate and putamen and 81.18% for amygdala, caudate, hippocampus, lateral ventricle, pallidus, and putamen. Although the experiments of these studies were conducted on different datasets, the relatively large improvement in results with our method indicates the advantage of using multiple atlases [see also other multi-atlas segmentation papers such as (Asman and Landman, 2013)].

The dataset used in our experiment is from the MICCAI 2012 Grand Challenge on Multi-atlas Labeling. The multi-atlas-based segmentation approaches were developed by different groups participated in the Grand Challenge. The segmentation results of subcortical structures from the various participants ranged from DSC 83.77–78.64%. While AutoSeg did not participate in the Grand Challenge, we computed the same measurements as employed in the challenge. AutoSeg achieves a segmentation performance of 81.73% of DSC, which places it within the upper mid-rank of the competition with ranking 5 out of 25 methods. The winning method (Wang et al., 2012) of the competition employed local similarity based weighting, whereas our current method employs global weighting computations. We plan to extend our method to include similar local weighting scheme.

As mentioned above, the local weighting algorithms achieved the best segmentation results in the MICCAI 2012 Grand Challenge on Multi-atlas Labeling (Wang et al., 2012; Asman and Landman, 2013). There is no conflict between “atlas selection” and “local weighting” based label fusion. Rather, the atlas selection algorithm we proposed in this paper can be combined with local weighting to improve performance; such an implementation is currently being added to AutoSeg.

Because of the large shape and intensity variations of the brain structures caused by disease, we need a database whose size is large enough to represent the variations of the data. Although the current atlas population (15 MRI scans) in our experiment is of limited size, our method has been shown to provide segmentation results at an acceptable performance level on the separate testing dataset (20 scans). In our current neuroimaging studies, we employ all training and testing datasets (totally 35 scans) for multi-atlas segmentation to improve the performance even further than reported here.

We select neighboring templates via an atlas clustering technique. As shown in Figure 4, the clusters are determined via overlapping paths between the atlases and the subject image. It is noteworthy that this overlap varies across subject images and thus the clustering needs to be recomputed for each image. Furthermore, per cluster, we choose the atlas closest to the subject to ensure the highest similarity for label fusion, while reducing the sample bias of choosing one atlas per cluster.

In the atlas selection step, the path computation was in part determined via an intensity-based similarity measure (mean square difference). As Rohlfing mentioned (Rolhfing, 2012), the intensity-based image similarity metric is not optimal to evaluate registration accuracy. However, the atlas selection was also determined by a deformation field-based shape similarity measure (Harmonic Energy). Furthermore, the purpose of the energy function we used for the atlas selection is to measure the similarity between two images but is not focused on evaluating the accuracy of registration. In addition, our intensity similarity is based on intensity-calibrated images, thus reducing potential confounding effects between the atlas selection and the image registration.

As mentioned above, skull-stripping is a critical step for brain segmentation. We used the ABC based brain tissue classification to identify GM, WM, and CSF and create the brain mask for the skull-stripping. We often run multiple iterations of ABC (this number of iterations is a parameter of AutoSeg) and use the skull-stripped ABC output from prior iterations as the initialization for the next iteration to improve the performance of the classification and skull-stripping. In the experiments of this study, ABC was iterated twice and thus the first iteration performed registration and tissue classification on a non-skull-stripped image, whereas the second iteration performed registration and tissue classification on the skull-stripped data from the first iteration.

We employ several existing tools/algorithms in the AutoSeg framework. Decisions on algorithms are based on (i) performance in our own tests as well as in the literature, and (ii) availability as open source. In general, AutoSeg employs the state-of-the-art tools in the field and each component has been vetted in our studies. All tools employed by AutoSeg are currently up-to-date (N4, ABC) or continuously being improved (BRAINSfit, ANTS, etc.). ANTS is the core registration tool employed and has consistently been shown to be the best current option for deformable registration (Klein et al., 2009). It is noteworthy that AutoSeg has a very flexible computational scheme that allows a developer to efficiently replace one component with a new tool, i.e., additional algorithms for different purposes can be easily added to AutoSeg.

Most of the recently developed multi-atlas segmentation algorithms including the STAPLE-based algorithms and the ANTS joint fusion algorithm participated in the 2012 Grand Challenge. In this paper, AutoSeg was tested on the same dataset of the Grand Challenge. The segmentation results of the subcortical structures were compared to the segmentation results of other algorithms that participated in the Grand Challenge and we found that AutoSeg would be ranked 5 out of 25. This result indicates that AutoSeg can provide good segmentation results that are comparable to other widely used multi-atlas segmentation methods. Furthermore, AutoSeg has a unique user friendly GUI. Thus, users without any computer science or technology background can also easily use AutoSeg in their studies.

The pipeline scripting by AutoSeg employs BatchMake, which is a cross-platform tool for batch processing large amounts of data. BatchMake scripts can be easily edited with any text editor or a specified BatchMake script editor developed by Kitware Inc. BatchMake is easy to use, and allows straightforward integration of the scripts into condor and SGE grid environments.

In datasets where large morphological or intensity changes are present [due to pathology, e.g., in Parkinson's disease, MPS, ALD, Duchenne muscular dystrophy (DMD)], and where AutoSeg is being applied, atlas selection has significant advantages as AutoSeg reduces the influence of common disproportion of the normative vs. pathology exhibiting atlases in such settings. On the other hand, selecting the best atlas in each cluster ensures that the label fusion is achieved from the atlases similar to the target, with the variability represented by the atlas population still being incorporated by the label fusion procedure. AutoSeg was tested on the same dataset from the MICCAI 2012 Grand Challenge on Multi-atlas Labeling, and it would be ranked 5th out of the 25 algorithms in the challenge. This result shows that AutoSeg can provide good segmentation results that are comparable to other widely used multi-atlas segmentation methods, with still room for improvement.

The segmentation of brain structures, in general, includes the segmentation of subcortical structures and cortical regions/parcellations. The proposed AutoSeg software pipeline allows for the direct labeling of cortical regions via standard atlas/multi-atlas-based segmentation. It further allows the combination of the tissue classification with the cortical regions for a joint classification/multi-atlas-based cortical parcellation.

The experimental results presented here illustrate the power of our multi-atlas AutoSeg MRI segmentation software pipeline. This software pipeline is publicly disseminated as open source on the NIH Neuroimaging Informatics Tools and Resources Clearinghouse (NITRC) website with accompanying testing datasets (http://www.nitrc.org/projects/autoseg).

## Conclusions

In conclusion, we have presented a multi-atlas segmentation scheme implemented in our comprehensive AutoSeg segmentation software pipeline. Graph based clustering is employed to select the closest atlas per cluster for a weighted label fusion procedure. We validated this method on a publicly available dataset. The results show that the proposed method achieved comparable segmentation results to other existing multi-atlas segmentation methods for subcortical structures. Overall, AutoSeg provides the field of brain MRI studies with an automated multi-atlas segmentation software pipeline for brain MRI neuroimaging studies.

## Author contributions

Jiahui Wang, Clement Vachet, Sylvain Gouttard, Clémentine Ouziel, and Emilie Perrot are the main developers of the AutoSeg segmentation software pipeline. Ashley Rumple has been significantly involved in the testing, debugging data preparation and English language editing of the paper. Guangwei Du and Xuemei Huang provided advice about the development of AutoSeg from a clinical perspective. Guido Gerig and Martin Styner were overseeing the entire project.

### Conflict of interest statement

The authors declare that the research was conducted in the absence of any commercial or financial relationships that could be construed as a potential conflict of interest.

## Appendix parameter settings of autoseg for experiments

The parameter settings are very important for users to implement a software tool (Tustison et al., 2013). The parameter settings of AutoSeg used in the Experiments can be found online on the NITRC website of AutoSeg, at http://www.nitrc.org/projects/autoseg. The settings were recorded in two text files: AutoSeg_Computation.txt and AutoSeg_Paramters.txt. The settings of input data directory, atlas directory, output directory, etc. can be found in AutoSeg_Computation.txt. The parameters related to processing algorithms, such as N4 intensity inhomogeneity correction, ABC, ANTS, etc. can be found in AutoSeg_Paramters.txt. All parameters are further itemized below (all parameters identified as AutoSeg parameters can be edited within the AutoSeg GUI and are stored in the parameter files):

1. Tissue Segmentation:
  - EM Software: ABC (default)
  - Filter Iterations: 10 (default)
  - Filter Time Step: 0.01 (default)
  - Filter Method: Curvature flow (default)
  - Max Bias Degree: 4 (default)
  - Initial Distribution Estimator: robust (default)
  - Prior 1: 1.3 (default)
  - Prior 2: 1.0 (default)
  - Prior 3: 0.7 (default)
  - Prior 4: 1.0 (default)
  - Prior 5: 0 (modified)
  - Fluid atlas warping: Warp (default)
  - Fluid Atlas Warp Iterations: 50 (default)
  - Fluid Atlas Warp Max Step: 0.1 (default)
  - Atlas Linear Mapping: affine (default)
  - Image Linear Mapping: id (default)
2. Registration to common space:
  - Registration: Rigid Registration (default)
  - Is ROIAtlasGridTemplate: 1 (default)
  - GridTemplate SizeX: 0 (default)
  - GridTemplate SizeY: 0 (default)
  - GridTemplate SizeZ: 0 (default)
  - GridTemplate SpacingX: 0 (default)
  - GridTemplate SpacingY: 0 (default)
  - GridTemplate SpacingZ: 0 (default)
  - Registration Initialization: useCenterOfHeadAlign (default)
  - Use T1 initial transform: 0 (default)
3. ANTS registration parameters:
  - Warping Method: ANTS (default)
  - ANTS Iterations: 100 × 50 × 25 (default)
  - ANTS CC weight: 1 (default)
  - ANTS CC region radius: 2 (default)
  - ANTS MI weight: 0 (default)
  - ANTS MI bins: 32 (default)
  - ANTS MSQ weight: 0 (default)
  - ANTS CC weight for 2nd modality: 0 (default)
  - ANTS CC region radius for 2nd modality: 0 (default)
  - ANTS MI weight for 2nd modality: 0 (default)
  - ANTS MI bins for 2nd modality: 0 (default)
  - ANTS MSQ weight for 2nd modality: 0 (default)
  - ANTS Registration Type: GreedyDiffeomorphism (default)
  - ANTS Registration Step: 0.25 (default)
  - ANTS Gaussian Smoothing: 1 (default)
4. Inhomoneity correction via N4:
  - N4 ITK Bias Field Correction: 1 (default)
  - N4 Number of iterations: 50,40,30 (default)
  - N4 Spline distance: 0 (default)
  - N4 Shrink factor: 4 (default)
  - N4 Convergence threshold: 0.0001 (default)
  - N4 BSpline grid resolutions: 1,1,1 (default)
  - N4 BSpline alpha: 0 (default)
  - N4 BSpline beta: 0.5 (default)
  - N4 Histogram sharpening: 0 (default)
  - N4 BSpline order: 3 (default)
5. Multi-Atlas
  - Label Fusion Algorithm: Weighted Majority Voting (default)
  - Intensity Energy Weight: 0.8 (modified)
  - Harmonic Energy Weight: 0.2 (modified)
